# Predictors of Overweight and Obesity in Early Care and Education Teachers during COVID-19

**DOI:** 10.3390/ijerph20032763

**Published:** 2023-02-03

**Authors:** Susan B. Sisson, Adrien Malek-Lasater, Timothy G. Ford, Diane Horm, Kyong-Ah Kwon

**Affiliations:** 1 Department of Nutrition Sciences, University of Oklahoma Health Sciences Center, 1200 N. Stonewall Ave, Oklahoma City, OK 73117, USA; 2 Department of Teaching, Learning, and Curriculum, College of Education and Human Services, University of North Florida, 1 UNF Drive, Jacksonville, FL 32224, USA; 3 Department of Educational Leadership and Policy Studies, The University of Oklahoma, 4502 E. 41st Street, 4W101, Tulsa, OK 74135, USA; 4 Early Childhood Education Institute, The University of Oklahoma-Tulsa, 4502 E. 41st Street, Tulsa, OK 74135, USA; 5 Department of Instructional Leadership and Academic Curriculum, University of Oklahoma, 820 Van Vleet, Norman, OK 73019, USA

**Keywords:** early childhood teachers, teacher well-being, obesity, teacher health, COVID-19, social-ecological model

## Abstract

The purpose of this cross-sectional study was to determine individual, sociocultural, policy, and economic predictors of overweight/obesity in early care and education (ECE) teachers to identify modifiable opportunities to enhance the health of this critical workforce. ECE teachers (n = 1434) in the U.S. completed an online survey in late spring to mid-summer 2020. Teachers self-reported height and weight; body mass index (BMI) and weight status were calculated. Teachers reported micro-environment variables including age, race, gender, obesogenic lifestyle behaviors, well-being, food security, personal health, stress, job stress, type of ECE, COVID-19 teaching modality, and age of children in the classroom. Logistic regression predicting overweight/obesity and linear regression predicting BMI were conducted. Teachers with more years of teaching experience (OR: 1.022: 95% CI 1.005, 1.039) and higher consumption of fast food (2.038: 1.310, 3.169) had higher odds of overweight/obesity. Teachers with higher levels of education (0.58: 0.407, 0.828) and higher physical health (0.836: 0.775, 0.902) had lower odds of overweight/obesity. Other variables were not associated with overweight/obesity. Variables significant in logistic regression were also associated with higher BMI. Additionally, Native American race (β = 2.467 SE = 1.206) and sedentary hours/day (β = 0.152 SE = 0.075) were associated with higher BMI. Implications for enhancing workplace health for these ECE teachers are emerging.

## 1. Introduction

In the United States, over 2 million early care and education (ECE) professionals provide care for over 10 million children [1]. The role of ECE teachers is critical, fulfilling several tasks beyond the primary functions of caring for, educating, and preparing young children for success in school [2]. Additional roles include supporting community economic development and stability by enabling families to work outside the home, earn wages, and engage in educational opportunities [3]. Equally important, yet under recognized even by ECE teachers themselves [4,5], is the role of promoting healthy lifestyle habits in young children [6]. Evidence demonstrates that ECE teachers have a critical role in supporting and encouraging the daily movement [7,8,9,10] and dietary intake [11,12,13,14] of young children.

Overweight and obesity has been identified as a global health concern [15]. ECE teachers have a prevalence of overweight and obesity that ranges up to 71–90% [16], which is overall higher than the U.S. national average of 74% [17] and the global prevalence of 33% [18]. Further, ECE teachers’ waist circumference, an important predictor of cardiometabolic disease, is between 105–106 cm [19,20], which surpasses health recommendations of ≤88 cm for women [21]. Obesogenic behaviors and obesity are serious concerns due to associations with elevated risk for diseases, such as diabetes and cardiovascular disease, and premature mortality [22,23,24].

Predictors of obesity are multi-level and complex [25,26,27,28] and include genetics factors in addition to lifestyle health behaviors such as diet, physical activity, and sleep [25,26,27,28,29,30,31]. Socio-economic characteristics also contribute to the prevalence of obesity and comprise income and income stability, social capital and cohesion, and social support and networks [25,26,27,28]. The physical and built environment in which an individual lives also impacts correlates of obesity regarding access to foods, or lack thereof, perceived and actual safety, occupation, and transportation-related physical activity [25,26,27,28]. Zubery et al. [32] identified that participants’, including school teachers, age, gender, marital status, years at work, sedentary behaviors, and transportation use significantly predict the overweight and obesity status.

Despite the importance of ECE teachers’ contribution to child health, development, and societal economic stability, the ECE workforce is at high risk for poor physical, psychological, and professional well-being [19,33], and the COVID-19 pandemic exacerbated the challenges [34]. ECE teachers feel undervalued and overstressed [35], earn low wages, [36], have limited access to health care [1], experience food insecurity at rates 3–4 times [35,37,38] higher than national averages, [39], are heavy users of public assistance programs such as Supplemental Nutrition Assistance Programs [36], and have numerous health issues and chronic illnesses [16].

Research documents that ECE teachers have obesogenic lifestyle behaviors that are associated with poor health outcomes. ECE teachers consume low amounts of fruits and vegetables [37,40] and high amounts of sugary beverages [19]. They also engage in insufficient physical activity and high volumes of sedentary behavior [19,41]. Furthermore, they report struggles with healthy food choices [4] and report barriers preventing going outdoors with children such as perceived uncomfortable weather, extra work with taking children outdoors, and low physical activity self-efficacy to engage actively with children [42]. Participation in obesogenic lifestyle behaviors is associated with poorer socioeconomic status (SES) and this low is attributed, in part, to causing excess weight gain [43]. While literature is scant describing the role of systematic racism in obesogenic behaviors, research does demonstrate systematic racism is a determinant of health [44] and associated with overweight and obesity [45]. As noted above, ECE teachers often earn low wages and may experience systemic racism and classism which impacts their ability to participate in health-enhancing behaviors.

During the COVID-19 pandemic, health concerns increased regarding obesity due to the increase in obesogenic behaviors [41] and the increased severity of COVID-19 symptoms and consequences in individuals with obesity [46]. Given the importance and vulnerability of this workforce, and the additional strains during COVID-19 pandemic, a greater understanding of the complex predictors of obesity is essential to guide interventions, of which there are currently few [16]. The purpose of this study is to determine predictors of overweight and obesity in a national sample of ECE teachers guided by an ecological framework to identify modifiable opportunities to enhance the health of this critical workforce. As noted above, few interventions exist for this workforce whose critical importance has recently been highlighted due to COVID-19.

## 2. Materials and Methods

### 2.1. Framework

Acknowledging the individual and contextual factors associated with obesity reported in the literature [47] in concert with the adoption of a whole teacher perspective [33] necessitates the application of a comprehensive framework to guide research and intervention such as The Analysis Grid for Environments Linked to Obesity (ANGELO). Briefly, ANGELO is a grid illustrating macro- and micro-environments and types of environments (physical, economic, policy, sociocultural, and individual) [48]. Macro-environments are supporting infrastructure such as policies, distribution chains, and economic influences impacting the micro-environments in which individuals spend time and engage in obesogenic or health-enhancing lifestyle behaviors. Micro-environments are those settings where groups of people may gather for specific purpose such as to shop, learn, eat, recreate, worship, etc. The ANGELO micro-environment framework (Table 1) guided the inclusion of specific predictors of obesity for this study. Variables described in Table 1 reflect those predictors included in these analyses, as well as other key variables within the respective micro-environments that were not available in this online survey but would be of interest for future research.

### 2.2. Participants and Design

A total of 1434 ECE teachers serving children from birth to age 5 (including Kindergarten) in 46 U.S. states completed a cross-sectional online survey in late spring to mid-summer of 2020, during the height of the COVID-19 pandemic shutdown. We included early childhood teachers working in a group care and education settings including family child care, child care centers, Early Head Start/Head Start centers, prekindergarten, public school. Teachers who work full-time were eligible to participate regardless of their demographic backgrounds. We also included teachers who tentatively do not work due to a center/school closure but are still affiliated with the center/closure. Teachers who completed the survey outside the U.S. were excluded from the study. Center and program directors were excluded from participation. For all but three variables included in the model, the value of missing data was under 1%. For obesity (and BMI) as well as sedentary hours per day, these numbers were 6 and 10%, respectively. The analytical sample size was 1106 based on listwise deletion of missing responses. However, Bonferroni-adjusted *t*-tests of missing data cases on all outcomes and independent predictors revealed no differences in the study variables between those with complete data and those with missing data on these three variables.

### 2.3. Procedures

After receiving Institutional Review Board approval from the University of Oklahoma, we recruited ECE teachers to complete an online survey via various social media platforms (e.g., Facebook, Twitter) and contacts with local, state, and national professional organizations (e.g., State Child Care Resource and Referral Services, National Head Start Association). To ensure responses from various states and types of settings, such as private childcare centers, public schools, Head Start programs, and family child care homes, stratified sampling (by state and setting type) was also integrated into the recruitment phase. This procedure involved producing a sample frame of ECE settings in each U.S. state and, from this, developing a contact list of family child care homes, child care centers, Head Start programs, and private and public schools which was first proportional to state population and then that sought to preserve U.S. representativeness by center type. We emailed directors of these centers to encourage their teachers to participate in the study. Other recruitment strategies, including a flyer on social media and using a list of teacher contacts who participated in the authors’ former research project, were also used. One hundred twenty teachers with complete surveys were randomly selected to receive a USD 50 electronic gift card.

**Measures and Instruments.** Our interdisciplinary research team used previously validated scales and, when none were available, developed questions related to teachers’ personal and professional backgrounds, teaching experiences, health, and well-being during the COVID-19 pandemic. The online survey took 25–30 min to complete. Participants were asked to report their heights and weights. Body Mass Index (BMI) was calculated by dividing weight in kilograms by the square of height in meters. BMI was further grouped into four weight status categories: ratios less than 18.5 were classified as underweight, between 18.5 and 24.9 as healthy, between 25 and 29.9 as overweight, and between 30 and 39.9 as obese [49].

**Individual Level.** Survey items at the individual level included demographic and personal descriptive characteristics such as age, race/ethnicity, gender, education level, and years working in ECE. Items also included personal health habits such as hours of sleep per night, changes in health due to COVID-19, physical activity, eating habits. Health status such as physical health, food security, and personal stress items were included. Hours of sleep was measured with a single question that we modified from an item from the National Health and Nutrition Examination Survey, asking participants to report, on an average night, how many hours of sleep they get per night, ranging from 1 h to 10 or more hours. To measure physical activity, questions asked how many days and hours participants spent on moderate-to-vigorous physical activities and how much time was spent sitting on a weekday, using elements of the International Physical Activity Questionnaire (IPAQ) Short Last 7 Days Self-administered format [50]. Time in moderate-to-vigorous physical activities was multiplied by days engaging in moderate-to-vigorous physical activities to determine total time in moderate-to-vigorous physical activity. Participants were categorized into two groups (≥150 min/week and <150 min/week) based on the U.S. Federal Physical Activity Guidelines [51]. Assessment of eating habits was informed by elements of the Project EAT-III survey [52] and asked about the frequency of eating breakfast, lunch, and fast food during a typical work week (i.e., 5 days). For meal skipping, participants were categorized by quartile, the lowest quartile being no more than 2 days per week and no more than 3 days per week, respectively. For frequency of eating fast food, participants were categorized by the quartile, the highest being eating fast food at least 3 days per week.

Overall physical health was assessed using the SF-12 Health Survey Standard, Version 1 [53], which is a commonly used instrument to measure health-related quality of life. The SF-12 consists of twelve items categorized in eight subscales (physical functioning, role physical, bodily pain, general health, vitality, social functioning, role emotional, and mental health). For this study, the physical component score (PCS) was used. Higher scores of the SF-12 PCS scales indicate a better health state with a score of 50 or higher indicating excellent physical health. Categories of perceived health are reported; however, continuous scores were used for analytical models. The United States Department of Agriculture 6-Item Short Form of the Food Security Survey Module [54] was modified for COVID-19 to include only 5 items. The score was adjusted accordingly. This questionnaire identifies food-insecure households where low scores of 0–1 indicate high or marginally good security, scores of 2–3 indicate low food security, and scores of 4–5 indicate very low food security. The Perceived Stress Scale [55] total score was the measure of perceived personal stress. This tool assesses how different situations affect a person’s feelings or perceived stress. Questions in the Perceived Stress Scale ask participants about feelings and thoughts during the last month (e.g., “How often have you felt nervous or stressed?”) and the frequency of their feelings ranging from 1 (rarely/never) to 5 (very often). For this study, total scores ranging from 1.0–2.3 indicate low stress, 2.4–3.6 indicate moderate stress, and 3.7–5.0 indicate high stress.

**Socio-Cultural Level**. Job stress at the socio-cultural level was assessed using a modified measure derived from the Job Content Questionnaire [56]. Our adapted measure consists of 11 items that measure three domains of stress: physical job demands (4 items), skill discretion (3 items), and decision authority (4 items). Each subscale is measured on a 5-point scale ranging from 1 (Strongly Disagree) to 5 (Strongly Agree) and is averaged across items yielding a range of 1–5. Participants rate how often each of the statements were true of their work in ECE settings. Higher scores in the job demand subscale indicate higher physical demand at work. Higher scores in the skill discretion indicate higher skill variety, and higher scores in the decision authority subscale indicate higher job control.

**Policy Level**. Items about the policy environment of the ECE setting in which participants worked were developed by the team and asked about the following potential predictors of obesity: whether the program was open or closed during the COVID-19 pandemic shutdown, the teaching modality during the shutdown, the type of program (community child care center, family child care home, Head Start, and public pre-k), and the social economic status (SES) of the population served at their program.

**Economic Level**. Participants self-reported whether or not they had health insurance and their annual salary to capture variables at the economic level of hypothesized predictors of obesity.

### 2.4. Analytical Approach

Analysis of survey data began with a descriptive analysis, and sample descriptive statistics are displayed in Table 2. Because the central question of the study was understanding the social and environmental predictors of weight status in ECE teachers, logistical regression analysis was used in tandem with a vector of covariates identified according to our ANGELO framework. All variables in the ANGELO framework (Table 1) were initially considered during analysis, however, due to issues of multicollinearity, the variables of teacher age, teacher gender, and type of center (community-based center, family child care home, Head Start, and public pre-k) were not included in the final analysis. To supplement this analysis, and to triangulate our results on the classification of overweight or obesity analysis, we also conducted an ordinary least squares (OLS) regression analysis of BMI using the identical set of predictors.

## 3. Results

The overall racial/ethnic composition of the sample is similar to ECE teachers nationally [1], with a slightly higher percentage of teachers self-identifying as Hispanic. The sample includes 56% White, 20% Hispanic, 14% Black, 6% Asian/other, and 4% Native American Indian or Alaska Native participants (Table 2). Fifteen percent of teachers reported they received public support including Medicaid, food stamps, or childcare subsidies. Teachers had a variety of educational backgrounds and training: 46.9% of participating teachers have less than a bachelor’s degree (7.5% high school education or less; 20.0% some college but no degree; 19.4% an associate degree) and 53.1% have a bachelor’s degree or higher (39.5% bachelor’s degree; 13.6% a graduate degree). Most teachers in the sample were women (98.3%). The average age of the participants was 42 (*Range =* 17 to 80).

Table 2 displays the individual, socio-cultural, policy, and economic characteristics of our sample of ECE teachers. Most striking is that 77% of our ECE teachers, based upon BMI, were classified as overweight or obese. According to teachers’ responses on obesogenic behaviors, only 39% of teachers reported exercising the recommended minimum of 150 min per week and had an average of 6.5 h of sedentary time per day. Twenty-seven percent of teachers skipped breakfast at least three days per week; 26% skipped lunch at least four days per week; and 26% our sample of teachers ate fast food at least three days per week. Perceived personal stress was 2.57 on a scale of 0–5, thus reporting moderate stress. Physical job demands were 2.9, skill discretion was 3.9, and decision authority was 3.4 each on a scale of 1–5. This indicates that ECE teachers in the sample experience a moderate level of physical job demands and decision authority (i.e., job control), but a moderate to high level of skill discretion, which means that their job often requires high-level skills that may often demand them to learn new things. Fifty-seven percent of teachers reported their program was open during the COVID-19 pandemic, but only 27% were teaching in-person during that time. The majority of respondents (76%) were preschool, pre-K, and kindergarten teachers and the remainder were infant and toddler teachers. Almost a quarter of the participating teachers had an annual income less than USD 20,000/year, but 88% had employer-provided health insurance during the pandemic.

The question of which predictors were associated with overweight/obese status was investigated via logistic regression (see Table 3). The overall model McFadden R^2^ is 0.109. Beginning with teacher individual-level characteristics, these results show a null effect for teacher race/ethnicity and number of children at home, but a negative effect for teachers’ degree attainment, such that teachers with a bachelor’s degree are nearly 50% less likely to be in the overweight/obese category than those who do not have a bachelor’s degree, *OR* = 0.58, 95% CI 0.41, 0.83, *p* < 0.01. Furthermore, teachers’ years of ECE experience was positively associated with overweight/obese status with every additional year of experience increasing their odds of being overweight/obese by 2.2%, *OR* = 1.02, 95% CI 1.01, 1.04, *p* < 0.01. With respect to teachers’ physical and health-related outcomes, after controlling for whether or not a teacher’s school was open and if they were teaching in person, we found null effects on physical exercise and sedentary behavior, *OR* = 0.77, 95% CI, 0.57, 1.04, *p = n.s.; OR* = 1.04, 95% CI 0.99, 1.09, *p = n.s*., respectively. However, teacher overall physical health was associated with lower odds of being overweight/obese, *OR* = 0.84, 95% CI 0.76, 0.90, *p* = 0.01. Finally, teachers’ fast food consumption was associated with their odds of being classified as overweight/obese, with teachers who ate fast food more than three days a week being over 2.0 times more likely to be overweight/obese, *OR* = 2.04, 95% CI 1.31, 3.17 *p* = 0.01. Self-reported sleep, negative changes in psychological well-being due to the pandemic, and frequency of eating breakfast or lunch were not significantly associated with overweight/obesity weight status.

Socio-cultural-level predictors such as physical job demands, decision making authority, and skill discretion were not associated with status of overweight or obesity. Similarly, policy-level variables such as if the program was open during COVID-19, teaching in-person, socio-economic status (SES) of students, and age of children in the classroom were not associated with overweight or obese classification. With regards to economic-level predictors, there was a null effect for teacher salary and for those who had employer-provided health insurance. Results for our OLS analysis of BMI were similar (see Table 4). An important difference observed in the linear model but not the primary logistic model was the relationship of sedentary behavior and higher BMI. In the linear regression, number of sedentary hours/day was positively associated with higher BMI, β = 0.15, SE = 0.08, *p* < 0.05; however, as noted above, there was not a significant association between sedentary hours and overweight or obesity classification. The overall model McFadden R^2^ is 0.129.

## 4. Discussion

The purpose of this study was to determine multi-level, ecological predictors of ECE teacher overweight and obesity status during an early phase of the COVID-19 pandemic. ECE teachers have a higher prevalence of overweight and obesity [19] and influence children’s health behaviors [7,8,9,10,11,12,13,14,57,58] and weight status [57,58]. A total of 77% of teachers in this study were classified as overweight or obese, which is slightly higher than the national averages of 69% of women 20–74 years as reported from the National Health and Nutrition Examination Survey [17]. It is important to note that this study was conducted during the early months of the COVID-19 pandemic shutdown and increases in body weight are a demonstrated effect of the shutdown [59]. However, our results are consistent with previous reports that the prevalence of overweight and obesity is higher in early childhood teachers than national samples [19].

A holistic approach or one examining multiple ecological levels to understand and intervene to promote healthy weight is needed to address the complexity of obesogenic behaviors and the ability to develop impactful interventions [60]. Predictors represented the individual-, socio-cultural-, policy-, and economic-level characteristics of the ANGELO framework [48]. At the individual level, we observed that teachers with more years of teaching experience and higher consumption of fast food were associated with higher odds of being overweight or obese. We also observed that teachers with higher levels of education and higher physical health were associated with lower odds of being overweight or obese. Individual-level variables such as sleep, meal skipping, food insecurity, and exercise were not associated with overweight/obesity status. Sedentary behavior was not associated with odds of overweight and obesity classification; however, in the secondary linear analyses, hours per day of sedentary behavior was associated with higher BMI. Other individual-level characteristics were not associated with overweight or obesity. Nearly half (44%) of teachers were of minority status and this was not associated with weight status. Teachers reported 14 years of experience and 53% have a bachelor’s degree or higher. Higher educational attainment was associated with weight status in alignment with previous literature [61]. The prevalence of teachers in our sample more closely aligns with previous research studies [62] that include early childhood educators teaching in environments which may require higher degree attainment, such as public school systems [63] compared to community-based child care.

We found that the fast food consumption is positively associated with overweight and obesity in our study. A quarter of the teachers eat fast food at least three days per week, which is less than nationally representative prevalence of eating fast food at least two times per week of 34.5% [64]. It is worth noting that frequency of fast food consumption decreased during the pandemic [65]. Frequent consumption of fast food is associated with increased energy intake [66] and increased 60–80% higher odds of obesity [31], supporting the findings in our study. Approximately one-third (36%) of teachers reported very good or excellent physical health. On average, teachers had lower scores of physical health according to the SF-12 PCS. This aligns with what the literature says about teachers’ obesogenic behaviors and health even before the pandemic [4,19,37,40]. One study reported that the rate of very good or excellent among ECE teachers dropped from 70% to 37% during COVID-19, which indicates the potential negative impact of the pandemic on teachers’ general physical health status [34]. The combination of low physical activity and a high volume of sedentary time increases risk for obesity, morbidity, and mortality [67]. The prevalence of teachers self-reporting that they attained the federally recommended 150 min per week of physical activity was lower than national averages (39% vs. 53%) [68]. Teachers reported 6.5 h of sedentary behaviors per day, which is similar to the national average of sitting time in Americans [59]. In our logistic regression analyses, neither physical activity nor sedentary behavior were associated with overweight and obesity, contrary to other studies [26,65]. However, in our linear analyses, hours of sedentary behaviors was associated with higher BMI, which is congruent with other literature [32].

The majority of teachers (62%) reported sleeping at least seven hours a night. National Sleep Guidelines state that healthy adults sleep between 7–9 h per night [69]. Nationally, 65.2% of adults in the U.S. accumulate sufficient sleep [70], which is similar to the participants in our study. However, many adults (40.7%) report negative sleep habit changes as a result of the pandemic [71]. While sleep was not associated with obesity in our study, this relationship has been demonstrated in other cross-sectional and longitudinal studies [26,30]. On average, teachers in our study reported moderate levels of perceived personal stress based on the perceived personal stress scale, which helps to understand how different situations affect feelings and perceived stress by asking about feelings and thoughts in the last month. Perceived stress was not a significant predictor in our analyses. However, our findings are aligned with previous findings on the challenges in teachers’ health and well-being during the pandemic [34,72]. While some studies indicate higher perceived stress is associated with higher levels of overweight and obesity [73,74], other studies suggest this relationship is not so clear cut and can also demonstrate inverse associations [75,76].

Over a quarter of teachers report regular meal skipping of breakfast and/or lunch, which is similar to national data. The National Health and Nutrition Examination Survey indicates that 24% of Americans skip breakfast and 19% skip lunch [77]. Meal skipping, especially breakfast, has been associated with overweight and obesity [29], although they were not associated in our study. The mean food insecurity score for these participants indicates marginal or high food security, which indicates this sample of teachers has higher food security than others [35]. Approximately a quarter of our sample was classified as experiencing food insecurity, which is less than previous reports of 31–35% [37,38]. We do not observe an association between food insecurity and weight status. These findings support some research studies [78] and contradict others [79]. The relationship between food insecurity and weight is not observed across all race/ethnic groups [79] and may likely be mediated by other dietary behaviors associated with limited access to food [78].

At the socio-cultural or policy levels, none of the predicted variables were associated with weight status. Overall, the teachers participating in this study demonstrated a moderate level of job demands and decision authority (i.e., job control). However, they experience moderate to high levels of skill discretion (e.g., my job requires a high level of skill), which is more likely to be applicable to teachers teaching online. This finding suggests that, although the job demands are likely to increase due to the sudden changes during the pandemic, it may not be directly associated with obesity. However, it is feasible that there is another mechanism through which the increased job demand would be associated with teachers’ health including obesity (e.g., being moderated by increased stress). Future studies are needed to explore other possibilities. At the policy level, none of the predicted variables were associated with weight status. Over half (57%) of the teachers’ schools were open and 27% were teaching in-person during the COVID-19 pandemic shutdown. Increases in body weight were observed during the COVID-19 shutdown [59] and the impact of schools remaining open and online vs. in-person teaching was uncertain.

None of the economic characteristics were associated with weight status. About 25% of the teachers reported annual incomes less than USD 20,000, which is lower than other reports of ECE teachers in child care contexts (39–42%) [19,20]. It would be expected that those teachers earning lower wages would have a higher prevalence of overweight and obesity based on these previously reported associations [25]. Given our study included both public school pre-k teachers in addition to child care and Head Start contexts, the annual income is likely higher [62], although wages may have been impacted throughout the COVID-19 shutdown. Data on wage continuation or cessation during the shutdown were not collected in this study. The majority of teachers (88%) had employer-provided health insurance during COVID-19, which is similar to other reports (90%) [35]. While this contradicts a previous study reporting a higher prevalence of overweight and obesity in those with public health insurance [80], the percentage of our teachers not receiving employer-provided health insurance was small and may have prohibited observation of a relationship. Generally, these factors are more distal and may be less likely to be associated with obesity than individual or more proximal factors. Further, the pandemic itself could impact both the social cultural and economic characteristics and their role in teachers’ well-being.

Findings of this study should be interpreted in light of their strengths, limitations, and contextual situation. Contextually, the current study utilizes this dataset to examine predictors of teachers’ weight status and does not specifically focus on COVID-19. However, it is relevant to note that these data were collected during the near complete shutdown early in the COVID-19 pandemic. Depending on a variety of factors and local/state regulations, some ECE programs continued or modified services, while others closed. The timing of this data collection is not necessarily a strength or limitation, but an important contextual consideration for generalization. Strengths include inclusion of a national sample of ECE teachers serving children from birth through age five years in multiple education and care contexts (i.e., community-based center, family child care home, Head Start, public school system). Further, the large sample size and multiple ecological levels of predictors collected allows for a thorough understanding of the multiple levels of influence on teachers’ weight status. While the majority of the conceptually relevant predictors were included in the parent study survey, some conceptually meaningful variables were not included. To this end, there were no variables included in the parent survey that represented physical aspects of the ECE environment.

Given this was a national survey, direct assessment of height, weight, or cardiorespiratory fitness was not feasible. With the numerous topics included in the survey and the need to minimize participant burden, some items in the survey are not examined at the depth which would offer greater insight, for example, detailed information on household size along with household income would have allowed the ability to determine the federal poverty level. Brevity in responses such as categorical responses and few questions on a given health behavior of interest potentially precluded the ability to detect associations. Furthermore, if this study was initially designed to address the relationship between environment and weight status in teachers, additional variables at each ecological level should be included. For example, aspects of the worksite that influence individual lifestyle behaviors were not included. Given that findings indicate the importance of these individual-level variables in this study, our study is limited by the ability to include this level of detail at the socio-cultural, policy, or economic levels. While self-report surveys can introduce social desirability bias, the value of collecting such multi-level data on such a large number of teachers across the U.S. outweighs the limitations of the rigor of measurement outside of online surveys. Previous research has also demonstrated that ECE teachers’ self-reported weight can be validated for its accuracy as it is similar to directly assessed weight [33]. Furthermore, this survey included a compilation of psychometrically strong survey instruments, which adds to its rigor.

Future directions from this emerging field of understanding the impact of the early care and education worksite broadly on teachers’ health and well-being and specifically on weight status are many. Future research should be designed to rigorously evaluate the individual contributors to weight status and weight gain and how environments supportive of healthy lifestyle behaviors promote health or disease. Current literature, including this study, has efficiently analyzed secondary data collected for other purposes. As such, rigor on specific measures associated with weight status limits the strength of the implications. Even with research on the impact of the early care and education worksite on teachers’ weight status in the early stages, interventional foundations are emerging. For instance, developing early care and education worksites in such a manner to promote the desired individual health behaviors, such as limited sedentary time, healthy dietary intake, and enhanced overall physical health, may promote healthy weight status. In our study, individual-level variables were associated with overweight/obesity and BMI. However, these behaviors do not occur in a vacuum and ECE teachers spend a great deal of time in the work environment. Individual- and systems-level interventions that foster healthy behaviors such as limiting meal skipping, fast food intake, and sedentary behavior, and promoting physical activity, healthy sleep habits, and stress management can be examined. The provision of free or low-cost breakfast, lunch, and even take-and-bake or ready-made dinners for early childhood teachers from their worksite may decrease meal skipping and fast food intake. Furthermore, this may work to promote food security, in a population known to have high levels of food insecurity [35,37,38]. Given the timing of this study occurring immediately after the COVID pandemic shutdown, relationships between predictors and obesity status may have been impacted in ways this study could not assess. Future studies can examine the role of the pandemic’s impact on social cultural and economic characteristics of early childhood teachers more broadly.

## 5. Conclusions

The purpose of this study was to determine multi-level, ecological predictors, guided by the ANGELO framework [48], of ECE teacher overweight and obesity status during an early phase of the COVID-19 pandemic. Similar to previous research on early childhood educators [4,19,37,40,41], the prevalence of overweight and obesity and obesogenic behaviors was high. Teachers with more time in the early childhood education industry, with higher consumption of fast food, and more time in sedentary behaviors (in the linear analyses only) were associated with higher odds of overweight and obesity. Some individual-level variables such as sleep, meal skipping, food insecurity, and exercise were not associated with overweight/obesity status. Social cultural, policy, and economic characteristics were not associated with overweight and obesity in this sample. With higher levels of overweight and obesity associated with longevity in a business that strives to ensure teacher stability and reduce turnover [81], the implication is clear that early care and education worksites need to develop cultures and practices that promote and support healthy individual lifestyle behaviors that will work to reverse the trajectory of weight gain. These system-level contributors to healthy lifestyle behaviors were not examined in this study and more research is warranted to determine if worksite in addition to individual-level intervention can reduce overweight/obesity status in ECE teachers.

## Figures and Tables

**Table 1:** Micro-Environment Predictors of Teacher Obesity within the ANGELO Framework.

ANGELO Levels	Micro-Environment Predictors Considered in This Study	Micro-Environment Predictors Not in This Study
Individual	Teacher age Teacher race/ethnicity Teacher gender Hours of sleep per night Change in health during COVID-19 pandemic Negative change in psychological well-being during COVID-19 pandemic Eating habits (consumption of breakfast, lunch, fast food) Food security Number of children at home Teacher education level Teacher years working in ECE Physical health (Personal overall general health rating) Exercise (days/hours vigorous physical activity, time spent sitting) Perceived personal stress	Cardiorespiratory fitness
Socio-cultural	Job Stress (Physical job demands, Skill discretion, Decision authority)	Having friends at work Support for well-being at work
Policy	Center/school open during COVID-19 pandemic Teaching modality (i.e., in-person, virtual) Type of program (e.g., Head Start, private) Age group of children in classroom (Infant/Toddler, Preschool, PreK, K, multi-age) SES of population served	Policies related to food served and teachers eating with children Policies related to physical activity/time to be outside Policies related to supporting well-being at work

ANGELO = Analysis Grid of Environments Linked to Obesity.

**Table 2:** Demographics and Well-being/Health of Surveyed ECE Teachers during the Early Pandemic (n = 1106).

Categories	Percentage OR Mean (*SD*)
Teacher Weight Status	
Currently Overweight or Obese	77%
Individual Teacher-Level Characteristics	
Teacher Race	
Black	14%
Hispanic	20%
Native American	4%
White	56%
Asian/Other	6%
Teachers with Bachelor’s degree or higher	53%
Teacher has at least one child at home	52%
Teacher ECE experience (years)	14.12 (9.78)
Sleeps at least 7 h/night	62%
Negative changes to psychological well-being during pandemic	48%
Skips breakfast at least 3 days/week	27%
Skips lunch at least 4 days/week	26%
Eats fast food at least 3 days/week	26%
Food insecurity (range 0–5) *	1.07 (1.44)
Prevalence of Food insecurity (low and very low food security)	25.7%
Physical health scale (SF-12, PCS) (range 6–26)	16.44 (2.49)
Physical health perceived excellent	6%
Physical health perceived very good	30%
Physical health perceived good	48%
Physical health perceived fair	14%
Physical health perceived poor	2%
Exercises more than 150 min a week	39%
Number of sedentary hours/day	6.50 (3.39)
Perceived personal stress (range 0–5)	2.57 (0.65)
Socio-Cultural-Level Characteristics	
Physical job demands (range 1–5)	2.91(0.82)
Skill discretion (range 1–5)	3.93 (0.69)
Decision authority range (1–5)	3.35 (0.69)
Policy-Level Characteristics	
Teacher’s school was open during COVID-19	57%
Teacher was teaching in-person during COVID-19	27%
Teacher works with mostly students from low SES	46%
Infant/Toddler Teacher (else Pre-K/K)	24%
Economic-Level Characteristics	
Teacher income less than USD 20k	23%
Teacher had employer-provided health insurance during COVID-19	88%

* Food insecurity scale scores: 0–1 indicate high or marginally good food security, scores of 2–3 indicate low food security, and scores of 4–5 indicate very low food security.

**Table 3:** Logistic Regression Predicting Overweight/Obese Weight Status for Early Care and Education Teachers (n = 1106).

	Odds Ratio for Overweight/Obese	*SE*	95% CI	
			Lower	Upper
Constant	65.936 **	75.477	6.994	621.580
Individual Teacher-Level Characteristics				
Race: Black	0.861	0.208	0.536	1.384
Race: Hispanic	1.199	0.260	0.784	1.835
Race: Native American	1.821	0.799	0.770	4.304
Race: Asian/Other	0.634	0.196	0.346	1.163
Bachelor’s degree earned BA/BS or higher	0.580 **	0.105	0.407	0.828
Teacher has at least one child at home	1.298	0.201	0.959	1.757
Teacher years of ECE experience	1.022 *	0.009	1.005	1.039
Sleeps at least 7 h/night	0.769	0.129	0.553	1.069
Neg. changes to psych. well-being in pandemic	1.139	0.196	0.814	1.595
Skips breakfast at least 3 days/week	0.735	0.127	0.523	1.032
Skips lunch at least 4 days/week	1.167	0.218	0.809	1.683
Eats fast food at least 3 days/week	2.038 **	0.459	1.310	3.169
Food insecurity	1.070	0.065	0.951	1.205
Physical health scale (SF-12, PCS)	0.836 **	0.032	0.775	0.902
Exercises more than 150 min/week	0.768	0.119	0.567	1.040
Number of sedentary hours/day	1.041	0.025	0.992	1.092
Perceived personal stress	0.762	0.108	0.578	1.005
Socio-Cultural-Level Characteristics				
Physical job demands	0.922	0.089	0.764	1.113
Decision authority	1.004	0.120	0.794	1.270
Skill discretion	1.046	0.127	0.825	1.326
Policy-Level Characteristics				
Teacher’s school was open during COVID-19	1.105	0.189	0.790	1.544
Teacher was in-person during COVID-19	0.783	0.172	0.509	1.204
Teacher works with mostly low SES students	1.208	0.196	0.879	1.660
Infant/Toddler Teacher else Pre-K/K	1.053	0.202	0.724	1.533
Economic-Level Characteristics				
Teacher income less than USD 20k	0.981	0.203	0.654	1.473
Employer provided insurance during pandemic	1.584	0.388	0.980	2.558

Note. Coefficients of Overweight/Obese are odds ratios. Teacher race: White was the reference group. BA/BS = Bachelors of Arts/Bachelors of Science (i.e., undergraduate degree); ECE = Early Care and Education; SES = Socioeconomic status; Pre-k = prekindergarten—kindergarten; neg. = negative; psych. = psychological ** *p* < 0.01, * p < 0.05.

**Table 4:** Ordinary Least Squares Regression Results Predicting Body Mass Index (BMI) in ECE Teachers (n = 1106).

	Β (BMI)	*SE*	95% CI	
			Lower	Upper
Constant	43.815 **	3.523	36.903	50.727
Individual Teacher-Level Characteristics				
Teacher race: Black	−0.138	0.790	−1.689	1.413
Teacher race: Hispanic	0.268	0.685	−1.076	1.613
Teacher race: Native American	2.467 *	1.206	0.101	4.834
Race: Asian/Other	−1.226	1.074	−3.333	0.881
Bachelor’s degree earned BA/BS or higher	−1.837 **	0.568	−2.952	−0.722
Teacher has at least one child at home	0.786	0.503	−0.201	1.774
Teacher years of ECE experience	0.047	0.027	−0.005	0.099
Sleeps at least 7 h/night	−0.331	0.530	−1.370	0.709
Neg. changes to psych. well-being in pandemic	0.210	0.552	−0.873	1.292
Skips breakfast at least 3 days/week	−0.905	0.567	−2.017	0.207
Skips lunch at least 4 days/week	1.035	0.592	−0.126	2.197
Eats fast food at least 3 days/week	3.227 **	0.624	2.002	4.452
Food insecurity	−0.153	0.183	−0.512	0.207
Physical health scale (SF-12, PCS)	−0.873 **	0.110	−1.089	−0.657
Exercises more than 150 min/week	−0.962	0.512	−1.966	0.042
Number of sedentary hours/day	0.152 *	0.075	0.005	0.300
Perceived personal stress	−0.516	0.458	−1.415	0.383
Socio-Cultural-Level Characteristics				
Physical job demands	−0.111	0.311	−0.720	0.499
Decision authority	−0.025	0.391	−0.792	0.743
Skill discretion	0.451	0.388	−0.311	1.213
Policy-Level Characteristics				
Teacher’s school was open	0.092	0.552	−0.992	1.175
Teacher was teaching in-person	0.422	0.703	−0.958	1.802
Teacher works with mostly low SES students	0.881	0.525	−0.150	1.913
Infant/Toddler Teacher else Pre-K/K	−0.549	0.606	−1.739	0.641
Economic-Level Characteristics				
Teacher income less than USD 20 k	0.433	0.657	−1.723	0.857
Employer provided insurance during pandemic	1.238	0.815	−0.361	2.837

Note. Teacher race: White was the reference group. BA/BS = Bachelors of Arts/Bachelors of Science (i.e., undergraduate degree); ECE = Early Care and Education; SES = Socioeconomic status; Pre-k = prekindergarten—kindergarten; neg. = negative; psych. = psychological. ** p < 0.01, * p < 0.05.

## Data Availability

Data supporting reported results can be accessed by communicating by email with the corresponding author.
